# Conformable fractional Dirac system on time scales

**DOI:** 10.1186/s13660-017-1434-8

**Published:** 2017-07-10

**Authors:** Tuba Gulsen, Emrah Yilmaz, Sertac Goktas

**Affiliations:** 1 Department of Mathematics, Faculty of Science, Firat University, Elazig, 23119 Turkey; 2 Department of Mathematics, Faculty of Science and Letters, Mersin University, Mersin, 33343 Turkey

**Keywords:** 34N05, 26A33, 35Q41, time scale, conformable fractional derivative, Dirac system

## Abstract

We study the conformable fractional (CF) Dirac system with separated boundary conditions on an arbitrary time scale $\mathbb{T}$. Then we extend some basic spectral properties of the classical Dirac system to the CF case. Eventually, some asymptotic estimates for the eigenfunction of the CF Dirac eigenvalue problem are obtained on $\mathbb{T} $. So, we provide a constructive procedure for the solution of this problem. These results are important steps to consolidate the link between fractional calculus and time scale calculus in spectral theory.

## Introduction

Fractional calculus means differentiation and integration of a noninteger order. The idea of fractional calculus was introduced by Leibniz and L’Hopital in 1695. However, the study of noninteger order derivatives did not appear in the literature until 1819, when Lacroix [1] presented a definition of the fractional derivative based on the usual expression for the *n*th derivative of the power function. Within years the fractional calculus became a very attractive topic for mathematicians. Fractional calculus has many applications in science and engineering such as memory of a variety of materials, signal identification, temperature field problems in oil strata, diffusion problems, *etc*. (see [2–5]). Many different forms of fractional differential operators like the Grunwald-Letnikow, Riemann-Liouville, Hadamard, Caputo, Riesz and conformable ones have been presented (see [6–10]). Recently, researchers have started to deal with the discrete versions of fractional calculus benefitting from the theory of time scale (see [11–16]). For example, Benkhettou *et al.* [17] introduced the concept of the CF derivative of order *α* on $\mathbb{T}$. They explained all properties of the CF derivative on $\mathbb{T}$. The CF derivative of a function defined on $\mathbb{T}$ reduces to the Hilger derivative when $\alpha=1$. Before expressing the CF derivative of order $\alpha\in(0,1]$ on $\mathbb{T}$, we should give a historical development of time scale calculus.

Time scale calculus was first considered by Hilger [18] in 1988 in his doctoral dissertation under the supervision of Aulbach [19, 20] to unify difference and differential equations. However, similar ideas had been used before and go back at least to the introduction of the Riemann-Stieltjes integral which unifies sums and integrals. More specifically, $\mathbb{T}$ is an arbitrary, non-empty, closed subset of $\mathbb{R} $. Many results as regards differential equations carry over quite easily to related results for difference equations, while other results seem to be totally different in nature. The time scale calculus can be applied to any fields in which dynamic processes are described by discrete or continuous time models. So, it has various applications involving non-continuous domains like modeling of certain bug populations, chemical reactions, phytoremediation of metals, wound healing, and maximization problems in economics and traffic problems. In recent years, several authors have obtained many important results in different topics on $\mathbb{T}$ (see [21–24]). Although there are many studies in the literature on $\mathbb{T}$, very little work has been done as regards BVPs (see [25–33]). The work of combining fractional calculus and time scale calculus in spectral theory is much less extensive. To fill this gap, we consider below the CF Dirac eigenvalue problem on an arbitrary time scale.

Let us consider the CF Dirac eigenvalue problem

1.1 $$ L_{\alpha}y(t)=BT_{\alpha}\bigl(y(t)\bigr)+Q(t)y^{\sigma}(t)= \lambda y^{\sigma }(t),\quad0< \alpha\leq1,t\in \bigl[ \rho(a),b \bigr] =\mathbb{J}\cap\mathbb{T}, $$

with separated boundary conditions

1.2 $$\begin{aligned}& \eta y_{1}\bigl(\rho(a)\bigr)+\beta y_{2}\bigl(\rho(a) \bigr) =0, \end{aligned}$$

1.3 $$\begin{aligned}& \gamma y_{1}(b)+\delta y_{2}(b) =0, \end{aligned}$$

where

$$B=\left ( \textstyle\begin{array} [c]{c@{\quad}c}0 & 1\\ -1 & 0 \end{array}\displaystyle \right ) ,\qquad Q(t)=\left ( \textstyle\begin{array} [c]{c@{\quad}c}q(t) & 0\\ 0 & r(t) \end{array}\displaystyle \right ) , $$

and $\lambda>0$ is a spectral parameter, $y^{\sigma}=y(\sigma)$. Throughout this study, we assume that $q,r\in\mathbf{L}_{2}^{\alpha}\mathbb{J}$ are real-valued, continuous functions where

$$\mathbf{L}_{2}^{\alpha}\mathbb{J}= \biggl\{ f: \int _{\rho(a)}^{b}f(t)\Delta^{\alpha}t< \infty \biggr\} . $$

Here, $T_{\alpha}(y(t))$ indicates the CF derivative of the function *y* order *α* and $( \gamma^{2}+\delta^{2} ) {( \eta^{2}+\beta ^{2} ) \neq0}$. Moreover, $y(t,\lambda)= ( y_{1}(t,\lambda ) ,y_{2}(t,\lambda))^{\mathbf{T}}\in C ( \mathbb{J},\mathbb{R} ) $ denotes the eigenfunction of problem (1.1)-(1.3) where $C ( \mathbb{J},\mathbb{R} ) $ is the space of all continuous functions on $\mathbb{J}$ and **T** denotes the transpose. We want to look at to the classical spectral theory of Dirac system from a different perspective. Here, spectral properties and results on the solution of problem (1.1)-(1.3) will be discussed for the first time with this study. By setting $\alpha=1$ in (1.1)-(1.3), the problem reduces to the classical Dirac eigenvalue problem which includes the Hilger derivative [32]. In the case of $\mathbb{T}=\mathbb{R}$ and $\alpha=1$ in (1.1)-(1.3), we get the following classical Dirac system:

1.4 $$ \begin{aligned}[c] &y_{2}^{\prime} = \bigl( \lambda-q(t) \bigr) y_{1}, \\ &y_{1}^{\prime} = \bigl( -\lambda+r(t) \bigr) y_{2}. \end{aligned}$$

Equation (1.4) is known as the first canonic form of the Dirac system. The Dirac operator is the relativistic Schrödinger operator in quantum physics. It is a modern presentation of the relativistic quantum mechanics of electrons intended to make new mathematical results accessible to a wider audience. It treats in some depth the relativistic invariance of a quantum theory, self-adjointness and spectral theory, qualitative features of relativistic bound and scattering states, and the external field problem in quantum electrodynamics, without neglecting the interpretational difficulties and limitations of the theory. There are several studies about the classical Dirac system from many perspectives in the literature (see [34–45]).

Let us give a brief description of the structure of our study. In Section 2, we express some fundamental notations and definitions as regards CF calculus on $\mathbb{T}$. In Section 3, we prove some basic theorems for the CF Dirac system on $\mathbb{T}$. Using some methods, we get asymptotic estimates of the eigenfunction for the problem (1.1)-(1.3) in Section 4. Some conclusions are presented in Section 5.

## Methods

In this section, we want to recall notations, lemmas and theorems for CF calculus on $\mathbb{T}$. To give basic results for the problem (1.1)-(1.3), we should express some fundamental notions as regards time scale calculus. The next definitions are crucial for this theory. Forward and backward jump operators at $t\in\mathbb{T}$, for $t<\sup\mathbb{T}$, are defined as

$$\sigma(t)=\inf\{s\in\mathbb{T}:s>t\},\qquad\rho(t)=\sup \{s\in \mathbb{T}:s< t\},$$

where $\inf\phi=\sup\mathbb{T}$, $\sup\phi=\inf\mathbb{T}$ and *ϕ* indicates the empty set. Therewithal, *t* is left-dense, left-scattered, right-dense and right-scattered provided that $\rho (t)=t$, $\rho(t)< t$, ${\sigma (t)=t}$, $\sigma(t)>t$, respectively. The distance from an arbitrary element $t\in\mathbb{T}$ to the closest element on the right is called the graininess of $\mathbb{T}$ and is determined by $\mu(t)=\sigma(t)-t$, $\mu:\mathbb {T}\rightarrow[0,\infty)$. A closed interval on $\mathbb{T}$ is denoted by $[a,b]_{\mathbb{T}}=\{t\in\mathbb{T}:a\leq t\leq b\}$, where *a* and *b* are fixed points of $\mathbb{T}$ with $a< b$. We also need to explain $\mathbb{T}^{\kappa}$ along with the set $\mathbb{T}$ to express the Hilger derivative of a function. If $\mathbb{T}$ has a left-scattered maximum *m*, then $\mathbb{T}^{\kappa}=\mathbb{T}-\{m\}$. Otherwise, $\mathbb{T}^{\kappa }=\mathbb{T}$. For a function *h* defined on $\mathbb{T}$, $h^{\Delta}$ is called the Hilger derivative of *h*. $h^{\Delta}$ is equal to the usual derivative $h^{\prime}$ if $\mathbb{T}=\mathbb{R}$ and it is equal to the usual forward difference Δ*h* if $\mathbb{T}=\mathbb{Z}$. For more definitions and notations related to time scale calculus, we refer the reader to [21].

In 2016, Benkhettou *et al.* [17] defined the CF derivative of order *α* and its properties on $\mathbb{T}$ to generalize the Hilger derivative. Let $h:\mathbb{T}\rightarrow \mathbb{R} $, $t\in\mathbb{T}^{\kappa}$ and $\alpha\in(0,1]$. For $t>0$, one can define $T_{\alpha}(h)(t)$ to be the number provided it exists with the property that, given any $\varepsilon>0$, there is a *δ*-neighborhood $V_{t}\subset\mathbb{T}$ of *t* such that

2.1 $$ \bigl\vert \bigl[ h\bigl(\sigma(t)\bigr)-h(s) \bigr] t^{\alpha-1}-T_{\alpha }(h) (t) \bigl[ \sigma(t)-s \bigr] \bigr\vert \leq\varepsilon \bigl\vert \sigma(t)-s \bigr\vert , $$

for all $s\in V_{t}$. $T_{\alpha}(h)(t)$ is the CF derivative of *h*, order *α* at *t*. If $\alpha=1$ in (2.1), it reduces to the Hilger derivative on $\mathbb{T}$ [21]. Benkhettou *et al.* [17] introduced the main properties of the CF derivative of order *α* by the following lemmas.

### Lemma 2.1

[17]

*Let* $\alpha\in(0,1]$, $t\in \mathbb{T}^{\kappa}$ *and* $h:\mathbb{T}\rightarrow \mathbb{R} $ *be a function*. *The following features hold*:

(i) *If* *h* *is CF differentiable of order* *α* *at* $t>0$, *then* *h* *is continuous at* *t*.
(ii) *If* *h* *is continuous at* *t* *which is right*-*scattered*, *then* *h* *is CF differentiable of order* *α* *at* *t* *with*
  $$T_{\alpha}(h) (t)=\frac{h(\sigma(t))-h(t)}{\mu(t)}t^{1-\alpha}. $$
(iii) *If* *t* *is right*-*dense*, *then* *h* *is CF differentiable of order* *α* *at* *t* *if and only if* $\lim_{s\rightarrow t}\frac{h(t)-h(s)}{t-s}t^{1-\alpha}$ *exists as a finite number*. *In this instance*
  $$T_{\alpha}(h) (t)=\lim_{s\rightarrow t}\frac{h(t)-h(s)}{t-s}t^{1-\alpha}. $$
(iv) *If* *h* *is CF differentiable of order* *a* *at* *t*, *then* $h(\sigma (t))=h(t)+\mu(t)t^{1-\alpha}T_{\alpha}(h)(t)$.

### Lemma 2.2

[17]

*Let* $h,g:\mathbb{T}\rightarrow \mathbb{R} $ *be CF differentiable functions of order* *α* *at* $t\in\mathbb{T}^{\kappa}$. *Then*

(i) $T_{\alpha}(h+g)(t)=T_{\alpha}(h)(t)+T_{\alpha}(g)(t)$.
(ii) $T_{\alpha}(\lambda h)(t)=\lambda T_{\alpha}(h)(t)$, $\lambda\in \mathbb{R} $.
(iii) $T_{\alpha}(hg)(t)=T_{\alpha}(h)(t)g(t)+ ( h\circ\sigma ) (t)T_{\alpha}(g)(t)=T_{\alpha}(h)(t)(g\circ\sigma)(t)+h(t)T_{\alpha}(g)(t)$.
(iv) $T_{\alpha} ( \frac{h}{g} ) (t)=\dfrac{T_{\alpha}(h)(t)g(t)-h(t)T_{\alpha}(g)(t)}{g(t)(g\circ\sigma)(t)}$, *where* $g(t)(g\circ\sigma )(t)\neq0$.

Now, let us recall the definition of the *α*-CF integral. Let $h:\mathbb{T}\rightarrow \mathbb{R} $ be a regulated function [21]. Then the *α*-CF integral of *h* is defined by [17]

2.2 $$ \int h(t)\Delta^{\alpha}t= \int h(t)t^{\alpha-1}\Delta t. $$

The *α*-CF integral of *h* reduces to the classical CF integral which is given by Khalil *et al.* for $\mathbb{T}=\mathbb{R} $ and $\alpha=1$ [7]. Furthermore, we get the definition of the indefinite integral on $\mathbb{T}$ for $\alpha=1$ [21].

If the indefinite *α*-CF integral of *h* order *α* is denoted by

$$H_{\alpha}(t)= \int h(t)\Delta^{\alpha}t, $$

then the Cauchy *α*-CF integral of *h* is defined by [17]

$$\int _{a}^{b}h(t)\Delta^{\alpha}t=H_{\alpha}(b)-H_{\alpha}(a), $$

for all $a,b\in\mathbb{T}$.

## Some spectral properties of CF Dirac system on time scales

In this section, we give some important results for the CF Dirac system on $\mathbb{T}$. It is well known that (1.1)-(1.3) has only real eigenvalues and its eigenfunctions are orthogonal when $\mathbb{T}=\mathbb{R} $ and $\alpha=1$ [34]. The following results will generalize this basic consequences to the CF case for the problem (1.1)-(1.3). Let us firstly give a lemma to be used in the proofs of the main theorems.

### Lemma 3.1

*Let* $h,g:\mathbb{T}\rightarrow \mathbb{R} $ *be continuous functions*, $a,b\in\mathbb{T}$ *and* $\alpha\in(0,1]$. *Then*

(i) $\int _{a}^{b}T_{\alpha}(h)(t)g(t)\Delta^{\alpha}t=h(b)g(b)-h(a)g(a)-\int _{a}^{b}h^{\sigma}(t)T_{\alpha}(g)(t)\Delta ^{\alpha}t$.
(ii) $\int _{a}^{b}h(t)T_{\alpha}(g)(t)\Delta^{\alpha}t=h(b)g(b)-h(a)g(a)-\int _{a}^{b}T_{\alpha}(h)(t)g^{\sigma}(t)\Delta^{\alpha}t$.

### Proof

The proof can easily be obtained by using a similar procedure to [21]. □

### Theorem 3.2

*The CF Dirac operator* $L_{\alpha}$ *is selfadjoint on* $\mathbf{L}_{2}^{\alpha}\mathbb{J}$.

### Proof

Let $x(t)= ( x_{1}(t),x_{2}(t) ) ^{\mathbf{T}}$ and $y(t)= ( y_{1}(t),y_{2}(t) ) ^{\mathbf{T}}$ be solutions of CF Dirac eigenvalue problem (1.1)-(1.3). Then

$$\begin{gathered} L_{\alpha}x(t) =BT_{\alpha}\bigl(x(t)\bigr)+Q(t)x^{\sigma}(t)= \lambda x^{\sigma }(t), \\ L_{\alpha}y(t) =BT_{\alpha}\bigl(y(t)\bigr)+Q(t)y^{\sigma}(t)= \lambda y^{\sigma }(t).\end{gathered}$$

By considering the definition of the inner product on $\mathbf {L}_{2}^{\alpha }\mathbb{J}$ and the boundary conditions, we get

$$\begin{aligned} \langle L_{\alpha}x,y \rangle ={}& \int_{\rho ( a ) }^{b} \bigl( L_{\alpha}x ( t ) \bigr) ^{\mathbf{T}}y ( t ) \Delta^{\alpha}t \\ ={}& \int_{\rho ( a ) }^{b} \bigl( \bigl( T_{\alpha} \bigl( x_{2}(t) \bigr) +q ( t ) x_{1}^{\sigma}(t) \bigr) y_{1} ( t ) + \bigl( -T_{\alpha} \bigl( x_{1}(t) \bigr) +r ( t ) x_{2}^{\sigma}(t) \bigr) y_{2} ( t ) \bigr) \Delta^{\alpha}t \\ ={}& \int_{\rho ( a ) }^{b}T_{\alpha} \bigl( x_{2}(t) \bigr) y_{1} ( t ) \Delta^{\alpha}t- \int_{\rho ( a ) }^{b}T_{\alpha} \bigl( x_{1}(t) \bigr) y_{2} ( t ) \Delta^{\alpha }t \\ &+ \int_{\rho ( a ) }^{b} \bigl( q ( t ) x_{1}^{\sigma }(t)y_{1}(t)+r ( t ) x_{2}^{\sigma}(t)y_{2}(t) \bigr) \Delta^{\alpha}t \\ ={}&x_{2}(t)y_{1} ( t ) \vert _{\rho ( a ) }^{b}- x_{1}(t)y_{2} ( t ) \vert _{\rho ( a ) }^{b}- \int_{\rho ( a ) }^{b}x_{2} ( t ) T_{\alpha} \bigl( y_{1}(t) \bigr) \Delta^{\alpha}t \\ &+ \int_{\rho ( a ) }^{b}x_{1} ( t ) T_{\alpha} \bigl( y_{2}(t) \bigr) \Delta^{\alpha}t + \int_{\rho ( a ) }^{b} \bigl( q ( t ) x_{1}^{\sigma }(t)y_{1}(t)+r ( t ) x_{2}^{\sigma}(t)y_{2}(t) \bigr) \Delta^{\alpha}t \\ ={}& \int_{\rho ( a ) }^{b} \bigl( \bigl( T_{\alpha} \bigl( y_{2}(t) \bigr) +q ( t ) y_{1}^{\sigma}(t) \bigr) x_{1} ( t ) + \bigl( -T_{\alpha} \bigl( y_{1}(t) \bigr) +r ( t ) y_{2}^{\sigma}(t) \bigr) x_{2} ( t ) \bigr) \Delta^{\alpha}t \\ ={}& \int_{\rho ( a ) }^{b}x^{\mathbf{T}} ( t ) L_{\alpha}y ( t ) \Delta^{\alpha}t \\ ={}& \langle x,L_{\alpha}y \rangle, \end{aligned}$$

where $t\in\mathbb{J}$ is right-dense. This completes the proof. □

### Theorem 3.3

*All eigenvalues of the problem* (1.1)-(1.3) *are real*.

### Proof

Let *λ̅* be a complex eigenvalue and $\overline{y}(t,\lambda)= ( \overline{y}_{1}(t),\overline{y}_{2}(t) ) ^{\mathbf{T}}$ be an eigenfunction corresponding to the eigenvalue *λ̅* of the problem (1.1)-(1.3). Since *q* and *r* are real-valued functions and *η*, *β*, *γ* and *δ* are also real, we obtain

$$\begin{aligned} T_{\alpha} \bigl( y_{1} ( t ) \bar{y}_{2}^{\sigma} ( t ) -\bar{y}_{1} ( t ) y_{2}^{\sigma} ( t ) \bigr) ={}&T_{\alpha} \bigl( y_{1} ( t ) \bigr) \bar{y}_{2}^{\sigma} ( t ) +y_{1}^{\sigma} ( t ) T_{\alpha} \bigl( \overline{y}_{2}^{\sigma} ( t ) \bigr) \\ & -T_{\alpha} \bigl( \bar{y}_{1} ( t ) \bigr) y_{2}^{\sigma } ( t ) -\bar{y}_{1}^{\sigma} ( t ) T_{\alpha} \bigl( y_{2}^{\sigma} ( t ) \bigr) \\ ={}& \bigl( -\lambda+r(t) \bigr) y_{2}^{\sigma} ( t ) \bar{y}_{2}^{\sigma} ( t ) + \bigl( \bar{\lambda}-q(t) \bigr) \bar{y}_{1}^{\sigma} ( t ) y_{1}^{\sigma} ( t ) \\ & - \bigl( -\bar{\lambda}+r(t) \bigr) \bar{y}_{2}^{\sigma} ( t ) y_{2}^{\sigma} ( t ) - \bigl( \lambda-q(t) \bigr) y_{1}^{\sigma } ( t ) \bar{y}_{1}^{\sigma} ( t ) \\ ={}&(\bar{\lambda}-\lambda) \bigl( y_{1}^{\sigma} ( t ) \bar{y}_{1}^{\sigma} ( t ) +y_{2}^{\sigma} ( t ) \bar{y}_{2}^{\sigma} ( t ) \bigr) \\ ={}&(\bar{\lambda}-\lambda) \bigl( \bigl\vert y_{1}^{\sigma} ( t ) \bigr\vert ^{2}+ \bigl\vert y_{2}^{\sigma} ( t ) \bigr\vert ^{2} \bigr) .\end{aligned}$$

If we take the *α*-CF integral of the last equality from $\rho ( a ) $ to *b* with respect to *t*, we have

$$(\bar{\lambda}-\lambda) \int_{\rho ( a ) }^{b} \bigl( \bigl\vert y_{1}^{\sigma} ( t ) \bigr\vert ^{2}+ \bigl\vert y_{2}^{\sigma } ( t ) \bigr\vert ^{2} \bigr) \Delta^{\alpha}t= \bigl( y_{1} ( t ) \bar{y}_{2}^{\sigma} ( t ) -\bar{y}_{1} ( t ) y_{2}^{\sigma} ( t ) \bigr) \big\vert _{\rho ( a ) }^{b}=0. $$

Since $\lambda\neq\bar{\lambda}$, we get $y_{1}^{\sigma} ( t ) =y_{2}^{\sigma} ( t ) \equiv0$. This is a contradiction. Hence, the eigenvalues of the problem (1.1)-(1.3) are real. □

### Theorem 3.4

*Let* $x= ( x_{1},x_{2} ) ^{\mathbf{T}},y= ( y_{1},y_{2} ) ^{\mathbf{T}}\in C_{\alpha }(\mathbb{J},\mathbb{R} )$ *be the eigenfunctions of the problem* (1.1)-(1.3). *Then we have*

- $( L_{\alpha}x ) ^{\mathbf{T}}y^{\sigma}- ( L_{\alpha }y^{\sigma} ) ^{\mathbf{T}}x^{\sigma}=T_{\alpha} ( W(x,y) ) $ *on* $\mathbb{J}\cap\mathbb{T}$.
- $\langle L_{\alpha}x,y^{\sigma}\rangle-\langle L_{\alpha}y^{\sigma},x^{\sigma}\rangle= W(x,y) \vert _{\rho ( a ) }^{b}$, *where* $W(x,y)=x_{2}y_{1}^{\sigma}-x_{1}y_{2}^{\sigma}$.

### Proof

Here $C_{\alpha}(\mathbb{J},\mathbb{R} )$ denotes the space of all functions whose CF derivatives of order *α* are continuous.

(a) The definition of *W* and the product rule for a CF derivative of order *α* yield

$$\begin{aligned} ( L_{\alpha}x ) ^{\mathbf{T}}y^{\sigma}- \bigl( L_{\alpha }y^{\sigma} \bigr) ^{\mathbf{T}}x^{\sigma} ={}& \bigl( T_{\alpha} \bigl( x_{2}(t) \bigr) +q ( t ) x_{1}^{\sigma}(t) \bigr) y_{1}^{\sigma } ( t ) - \bigl( T_{\alpha} \bigl( x_{1}(t) \bigr) -r ( t ) x_{2}^{\sigma}(t) \bigr) y_{2}^{\sigma} ( t ) \\ & - \bigl( T_{\alpha} \bigl( y_{2}^{\sigma} ( t ) \bigr) +q ( t ) y_{1}^{\sigma} ( t ) \bigr) x_{1}^{\sigma } ( t ) + \bigl( T_{\alpha} \bigl( y_{1}^{\sigma} ( t ) \bigr) -r ( t ) y_{2}^{\sigma} ( t ) \bigr) x_{2}^{\sigma} ( t ) \\ ={}&T_{\alpha} \bigl( x_{2}(t) \bigr) y_{1}^{\sigma} ( t ) -T_{\alpha} \bigl( x_{1}(t) \bigr) y_{2}^{\sigma} ( t ) -T_{\alpha} \bigl( y_{2}^{\sigma} ( t ) \bigr) x_{1}^{\sigma } ( t ) \\ &+T_{\alpha} \bigl( y_{1}^{\sigma} ( t ) \bigr) x_{2}^{\sigma} ( t ) \\ ={}&T_{\alpha} \bigl( x_{2}(t)y_{1}^{\sigma} ( t ) -x_{1}(t)y_{2}^{\sigma} ( t ) \bigr) \\ ={}&T_{\alpha} \bigl( W(x,y) \bigr) . \end{aligned}$$

(b) By using the inner product on $\mathbf{L}_{2}^{\alpha}\mathbb{J}$, we get

$$\begin{aligned} \bigl\langle L_{\alpha}x,y^{\sigma}\bigr\rangle - \bigl\langle L_{\alpha}y^{\sigma},x^{\sigma}\bigr\rangle & =\int _{\rho ( a ) }^{b} \bigl( ( L_{\alpha}x ) ^{\mathbf {T}}y^{\sigma}- \bigl( L_{\alpha}y^{\sigma} \bigr) ^{\mathbf{T}}x^{\sigma } \bigr) \Delta^{\alpha}t\\ & =\int_{\rho ( a ) }^{b}T_{\alpha} \bigl( W(x,y) \bigr) \Delta^{\alpha}t\\ & = W(x,y) \vert _{\rho ( a ) }^{b}. \end{aligned} $$

Hence, the proof is completed. □

### Theorem 3.5

*The eigenfunctions* $x(t,\lambda _{1})= ( x_{1}(t,\lambda_{1}),x_{2}(t,\lambda_{1}) ) ^{\mathbf{T}} $ *and* $y(t,\lambda_{2})= ( y_{1}(t,\lambda_{2}),y_{2}(t,\lambda _{2}) ) ^{\mathbf{T}}$ *of the problem* (1.1)-(1.3) *corresponding to distinct eigenvalues* $\lambda_{1}$ *and* $\lambda_{2}$ *are orthogonal on* $\mathbf{L}_{2}^{\alpha}\mathbb{J}$, *i*.*e*.

$$\int _{\rho(a)}^{b}x^{\mathbf{T}}(t,\lambda_{1})y(t, \lambda_{2})\Delta^{\alpha}t=0. $$

### Proof

Since $x(t,\lambda_{1})$ and $y(t,\lambda _{2})$ are solutions of the CF Dirac eigenvalue problem (1.1)-(1.3), we obtain

$$\begin{gathered} T_{\alpha}\bigl(x_{2} ( t,\lambda_{1} ) y_{1} ( t,\lambda _{2} ) -x_{1} ( t, \lambda_{1} ) y_{2} ( t,\lambda _{2} ) \bigr) \\ \quad =T_{\alpha}\bigl(x_{2} ( t,\lambda_{1} ) \bigr)y_{1} ( t,\lambda_{2} ) +x_{2}^{\sigma} ( t,\lambda _{1} ) T_{\alpha}\bigl(y_{1} ( t, \lambda_{2} ) \bigr) \\ \qquad{} -T_{\alpha}\bigl(x_{1} ( t,\lambda_{1} ) \bigr)y_{2} ( t,\lambda _{2} ) -x_{1}^{\sigma} ( t,\lambda_{1} ) T_{\alpha}\bigl(y_{2} ( t, \lambda_{2} ) \bigr) \\ \quad =\bigl(\lambda_{1}-q(t)\bigr)x_{1}^{\sigma} ( t, \lambda_{1} ) y_{1} ( t,\lambda_{2} ) +\bigl(- \lambda_{2}+r(t)\bigr)y_{2}^{\sigma} ( t,\lambda _{2} ) x_{2}^{\sigma} ( t,\lambda_{1} ) \\ \qquad{} -\bigl(-\lambda_{1}+r(t)\bigr)x_{2}^{\sigma} ( t, \lambda_{1} ) y_{2} ( t,\lambda_{2} ) -\bigl( \lambda_{2}-q(t)\bigr)y_{1}^{\sigma} ( t, \lambda_{2} ) x_{1}^{\sigma} ( t,\lambda_{1} ) \\ \quad = ( \lambda_{1}-\lambda_{2} ) \bigl[ x_{1}^{\sigma} ( t,\lambda_{1} ) y_{1}^{\sigma} ( t, \lambda_{2} ) +x_{2}^{\sigma} ( t,\lambda_{1} ) y_{2}^{\sigma} ( t,\lambda_{2} ) \bigr] , \end{gathered}$$

where *t* is right-dense. By taking the *α*-CF integral of the last equality from $\rho ( a ) $ to *b* with respect to *t*, we get

$$\begin{gathered} (\lambda_{1}-\lambda_{2}) \int_{\rho ( a ) }^{b} \bigl[ x_{1}^{\sigma} ( t,\lambda_{1} ) y_{1}^{\sigma} ( t, \lambda_{2} ) +x_{2}^{\sigma} ( t,\lambda_{1} ) y_{2}^{\sigma} ( t,\lambda_{2} ) \bigr] \Delta^{\alpha}t \\ \quad = \bigl[ x_{2} ( t,\lambda_{1} ) y_{1} ( t,\lambda _{2} ) -x_{1} ( t,\lambda_{1} ) y_{2} ( t,\lambda _{2} ) \bigr] \big\vert _{\rho ( a ) }^{b} \\ \quad =0, \end{gathered}$$

or

$$(\lambda_{1}-\lambda_{2}) \int _{\rho(a)}^{b}x^{\mathbf{T}}(t,\lambda _{1})y(t,\lambda_{2})\Delta^{\alpha}t=0. $$

Since $\lambda_{1}\neq\lambda_{2}$, it shows that $x(t,\lambda_{1})$ and $y(t,\lambda_{2})$ are always orthogonal. □

## Asymptotic estimates of eigenfunctions for CF Dirac system on time scales

In this section, we get the asymptotic estimates of the eigenfunction of the problem (1.1)-(1.3) on $\mathbb{T}$.

### Theorem 4.1

*The eigenfunction* $y(t,\lambda )= ( y_{1}(t,\lambda),y_{2}(t,\lambda) ) ^{\mathbf{T}}$ *is a solution of the problem* (1.1)-(1.3) *if and only if the component functions* $y_{1} ( t,\lambda ) $ *and* $y_{2} ( t,\lambda ) $ *satisfy the equations*

$$\begin{aligned} y_{1} ( t,\lambda ) ={}&\beta+\frac{\eta\lambda ( t^{\alpha }- ( \rho(a) ) ^{\alpha} ) }{\alpha}\\ &-\eta \int _{\rho (a)}^{t}r ( s ) \Delta^{\alpha}s-\lambda \int _{\rho(a)}^{t} \int _{\rho(a)}^{\sigma(s)} \bigl( \lambda-q ( u ) \bigr) y_{1}^{\sigma} ( u,\lambda ) \Delta^{\alpha}u \Delta^{\alpha}s \\ & + \int _{\rho(a)}^{t}r(s) \biggl( \int _{\rho(a)}^{\sigma (s)} \bigl( \lambda-q ( u ) \bigr) y_{1}^{\sigma} ( u,\lambda ) \Delta^{\alpha}u \biggr) \Delta^{\alpha}s \end{aligned}$$

*and*

$$\begin{aligned} y_{2} ( t,\lambda )={}&{-}\eta+\frac{\beta\lambda ( t^{\alpha }- ( \rho(a) ) ^{\alpha} ) }{\alpha}\\ &-\beta \int _{\rho (a)}^{t}q ( s ) \Delta^{\alpha}s+\lambda \int _{\rho(a)}^{t} \int _{\rho(a)}^{\sigma(s)} \bigl( -\lambda+r ( u ) \bigr) y_{2}^{\sigma} ( u,\lambda ) \Delta^{\alpha}u \Delta^{\alpha}s \\ & - \int _{\rho(a)}^{t}q(s) \biggl( \int _{\rho(a)}^{\sigma (s)} \bigl( -\lambda+r ( u ) \bigr) y_{2}^{\sigma} ( u,\lambda ) \Delta^{\alpha}u \biggr) \Delta^{\alpha}s, \end{aligned}$$

*respectively*.

### Proof

Let $y(t,\lambda)$ be a solution of the problem (1.1)-(1.3). Then the system of equations (1.1) is equivalent to the system

$$\begin{gathered} T_{\alpha}\bigl(y_{2}(t,\lambda)\bigr)+q(t)y_{1}^{\sigma} ( t,\lambda ) =\lambda y_{1}^{\sigma} ( t,\lambda ), \\ -T_{\alpha}\bigl(y_{1}(t,\lambda)\bigr)+r(t)y_{2}^{\sigma}( t,\lambda ) =\lambda y_{2}^{\sigma} ( t,\lambda ) . \end{gathered} $$

From this, we get

$$y_{2}(t,\lambda)=-\eta+ \int _{\rho(a)}^{t} \bigl( \lambda-q(s) \bigr) y_{1}^{\sigma}(s,\lambda)\Delta^{\alpha}s $$

and

$$y_{1}(t,\lambda)=\beta+ \int _{\rho(a)}^{t} \bigl( -\lambda+r(s) \bigr) y_{2}^{\sigma}(s,\lambda)\Delta^{\alpha}s. $$

By using the notions of $y_{1}^{\sigma}(t,\lambda)$ and $y_{2}^{\sigma}(t,\lambda)$ in the above equations, respectively, we obtain

$$\begin{aligned}& \begin{aligned} y_{2} ( t,\lambda ) ={}&{-}\eta+ \int _{\rho(a)}^{t} \bigl( \lambda-q ( s ) \bigr) \biggl( \beta+ \int _{\rho (a)}^{\sigma(s)} \bigl( -\lambda+r ( u ) \bigr) y_{2}^{\sigma } ( u,\lambda ) \Delta^{\alpha}u \biggr) \Delta^{\alpha}s \\ ={}&{-}\eta+\beta \int _{\rho(a)}^{t} \bigl( \lambda-q ( s ) \bigr) \Delta^{\alpha}s\\ &+ \int _{\rho(a)}^{t} \bigl( \lambda-q ( s ) \bigr) \biggl( \int _{\rho(a)}^{\sigma(s)} \bigl( -\lambda+r ( u ) \bigr) y_{2}^{\sigma} ( u,\lambda ) \Delta^{\alpha}u \biggr) \Delta^{\alpha}s \\ ={}&{-}\eta+\frac{\beta\lambda ( t^{\alpha}- ( \rho(a) ) ^{\alpha} ) }{\alpha}\\ &-\beta \int _{\rho(a)}^{t}q ( s ) \Delta^{\alpha}s+\lambda \int _{\rho(a)}^{t} \int _{\rho(a)}^{\sigma(s)} \bigl( -\lambda+r ( u ) \bigr) y_{2}^{\sigma} ( u,\lambda ) \Delta^{\alpha}u \Delta^{\alpha}s \\ & - \int _{\rho(a)}^{t}q(s) \biggl( \int _{\rho(a)}^{\sigma (s)} \bigl( -\lambda+r ( u ) \bigr) y_{2}^{\sigma} ( u,\lambda ) \Delta^{\alpha}u \biggr) \Delta^{\alpha}s, \end{aligned}\\& \begin{aligned} y_{1} ( t,\lambda ) ={}&\beta+ \int _{\rho(a)}^{t} \bigl( -\lambda+r ( s ) \bigr) \biggl( -\eta+ \int _{\rho (a)}^{\sigma(s)} \bigl( \lambda-q ( u ) \bigr) y_{1}^{\sigma } ( u,\lambda ) \Delta^{\alpha}u \biggr) \Delta^{\alpha}s \\ ={}&\beta-\eta \int _{\rho(a)}^{t} \bigl( -\lambda+r ( s ) \bigr) \Delta^{\alpha}s\\ &+ \int _{\rho(a)}^{t} \bigl( -\lambda+r ( s ) \bigr) \biggl( \int _{\rho(a)}^{\sigma(s)} \bigl( \lambda-q ( u ) \bigr) y_{1}^{\sigma} ( u,\lambda ) \Delta^{\alpha}u \biggr) \Delta^{\alpha}s \\ ={}&\beta+\frac{\eta\lambda ( t^{\alpha}- ( \rho(a) ) ^{\alpha} ) }{\alpha}\\ &-\eta \int _{\rho(a)}^{t}r ( s ) \Delta^{\alpha}s-\lambda \int _{\rho(a)}^{t} \int _{\rho(a)}^{\sigma(s)} \bigl( \lambda-q ( u ) \bigr) y_{1}^{\sigma} ( u,\lambda ) \Delta^{\alpha}u \Delta^{\alpha}s \\ & + \int _{\rho(a)}^{t}r(s) \biggl( \int _{\rho(a)}^{\sigma (s)} \bigl( \lambda-q ( u ) \bigr) y_{1}^{\sigma} ( u,\lambda ) \Delta^{\alpha}u \biggr) \Delta^{\alpha}s. \end{aligned} \end{aligned}$$

It completes the proof. □

## Conclusions

Fractional type eigenvalue problems have attracted the attention of many authors. Because of this, we consider a CF Dirac equation system with boundary conditions on $\mathbb{T}$ to obtain some spectral properties. Finally, we get asymptotic estimates of the eigenfunction for the problem (1.1)-(1.3). We know that these results are important steps for fractional spectral theory on time scales. As the work in this area progresses, we believe that many specific results will be obtained as regards this topic. For this purpose, this study will be very useful. As further work, we think the ideas can be extended to obtain asymptotic estimates of the eigenvalues for eigenvalue problems. After this stage, we can define inverse problem on time scales. Thus, further important results will be achieved.
